# NEWS 3

**DOI:** 10.7189/jogh.07.010203

**Published:** 2017-06

## Resources

### Demography

➢ Cuba’s economy has long been stunted by its isolation from the outside world, and its reliance on partners which have themselves collapsed (eg, the Soviet Union and Venezuela). Improving relations with the US had helped boost economic growth, but Venezuela’s economic crisis led to official figures showing a fall of 0.9% fall in Cuba’s GDP. Raul Castro, Cuba’s president has called for a more welcoming attitude to foreign investment and increased local production to replace imports to support the country’s economy. However, any attempts to boost economic growth could be arrested by Cuba’s stagnating population growth. Its population has reached a peak of 11 million, and is projected to fall due to an ageing population and a birth–rate below the replacement level. This has been exacerbated by improved US relations, as a wave of migrants left Cuba over the past 2 years, and many young Cubans plan to leave due to a lack of opportunities at home. This trend could intensify if the government pursues painful policies to counteract a contracting economy. (Business Insider, 7 January 2017)

➢ The percentage of UK women working into their 70s has doubled in the last 4 years, nearly reaching the same level as men, with 5.6% women stopping work after the age of 70 in 2012. This rose to 11.3% (or 150 000 women) in 2016 as increases in longevity, worries over pension income and desires to stay active has pushed up workforce participation rates among older women. An estimated 15.6% of men stopped working in their 70s in 2016. This trend is underpinned by legislation to end age discrimination and more flexible working patterns. Often, women have shortfalls in their pension provisions due to career breaks to raise children. Although continuing to work later in life is beneficial if by choice, employers risk demotivated and unproductive workforces if workers are forced to work by a lack of pension provision. Recently, the UK government launched a strategy to encourage the over–50s to consider a second career to extend their working lives. “Staying in work for a few more years can make a significant difference, not only to someone’s income but also their physical and mental health,” says Damian Green, the UK’s Work and Pensions Secretary. (BBC, 22 March 2017)

➢ Nearly 20 years ago, world leaders launched a new initiative to get new and underused vaccines to the world’s poorest people, at a time when millions of children died each year from vaccine–preventable diseases. This was the start of the GAVI Alliance, which has saved more than 7 million lives as child mortality has plummeted in most countries worldwide. Today, argues Dr Ngozin Okanjo–Iweala, the Chair of GAVI, the world is facing an equally acute need – 800 million children who do not have access to quality education. By 2030, many young people will lack basic secondary–school level skills, which are essential for 21^st^ century jobs. Young people lacking in skills may feel hopeless and turn to violence. Dr Okanjo–Iweala is a member of the Education Commission, a group of countries which works together to ensure that the world’s children are all in education – “vaccinating the world against poverty”. Their message is that quality education is the strongest antidote to poverty, and they have developed an innovative financing plan for education. This tool would leverage up to US$ 13 billion for education by 2030, working with development backs to coordinate funding and create financing packaging the multiply the impact of donor dollars to fill the funding up between governments and donors. This investment in human capital would strengthen families, communities and countries, making the world more stable and prosperous. (Thomson Reuters Foundation, 27 April 2017)

➢ For the first time in Canada’s history, there are more Canadian citizens aged over 65 years than under 15 years, and from 2011–16 the country had its largest ever–increase in the proportion of senior citizens. This data, from the 2016 census, shows that the number of senior citizens grew by 20%, against an overall 5% population increase. Canada’s population of children aged under 15 grew at 4.1%, reflecting lower fertility rates, which are now 1.6 children per woman. In line with increasing life expectancy – currently at 82 years – the number of centenarians grew by more than 40%. As women live longer than men, this increasing longevity means than among people aged over 85 years, there are two women for every man. This changing nature of Canada’s demography means that in just over 10 years, nearly 25% of Canadians will be aged over 65 years, with just 16% aged under 15, with resultant effects on tax revenue and health spending; and changing patterns of consumption and housing. While immigration has had a major impact on Canada’s population growth, it has had little effect on these trends, partly because immigration has been stable; and also because immigrants arrive as young adults and grow old in Canada. (National Post, 3 May 2017)

➢ According to the IMF, Asia’s changing demography means that the region is moving from being the biggest contributor to the global workforce, to subtracting millions of people from it. It estimates that population growth with fall to zero by 2050 (with Japan already experiencing negative growth), and that the percentage of working–age people has already hit its peak. This unravelling of Asia’s demographic dividend could hamper global and regional economic growth. The IMF highlights how Asia is undergoing accelerated aging compared to Europe and the USA, with aging happening at lower levels of per capita income compared to other developed countries. The speed of change hinders these countries from adapting to change, and they risk being “old before becoming rich.” The IMF estimates that Japan’s economy could shrink by 1% a year over the next 30 years as a result, and China, Hong Kong, South Korea and Thailand experiencing fall of between 0.5–0.75%, with an overall impact on the world’s economy of 0.1% each year. Labour market, pensions and retirement systems reform could help offset these adjustments, and the region’s working–age population could be boosted by immigration. The region also needs to improve productivity to offset slowing investment caused by excessive savings rates, the growth of less productive sectors (eg, services), and the declining importance of external trade. (Bloomberg, 7 May 2017)

### Economy

➢ From 2000 onwards, the economies of the world’s richest and poorer countries have begun to converge, as the latter undergo faster economic growth. Despite this welcome trend, many countries are left behind, including the world’s poorest, with the lowest – and diverging – average income growth. The emergence of these trends – convergence overall, but divergence among the poorest countries – is vital to understand global goals on the reduction of extreme poverty. This is shown by the success of the MDG in halving the rate of extreme poverty in developing countries – an example of convergence – even as the number of people living in extreme poverty in fragile states has risen, and the living standards of the very poorest people (the “consumption floor”) has barely risen. The poorest countries will face the biggest challenge in achieving the first SDG of ending extreme poverty everywhere. The World Bank has adopted a target rate of 3% for extreme poverty by 2030, but this will mask gaps in poverty rates, with a minority of countries having higher rates, and may continue to diverge and be left behind. There are 30 countries at risk of being left behind (including Malawi, St Lucia, and Zambia). 25 of these countries are relatively small, so risk being overlooked in the World Bank’s global poverty monitoring as the 3% target is compatible with several small countries failing to progress (although the Bank stresses the need to reduce poverty everywhere), and collectively they could account for more than 30% of all people living in extreme poverty – 280 million people. 18 out of the 30 are fragile, and 9 are resource–rich. (Brookings, 24 January 2017)

➢ In 2016, Zimbabwe’s central bank began printing a new form of money, the “bond note”, which is pegged at US$ parity. Zimbabwe adopted the US$ in 2009 after experiencing disastrous hyperinflation, but queues of citizens waiting to withdraw cash from banks reveal little faith in the government’s latest economic plans. Nearly 25% of Zimbabweans rely on food assistance, and 72% live in poverty – in a country with abundant natural resources, a once–booming agricultural sector and abundant human capital. However, much has been squandered during Robert Mugabe’s 37–year regime. Mr Mugabe began promisingly in 1980 following independence from Britain, with calls for reconciliation, better education and health care, but degenerated into oppressive and undemocratic rule, bringing the country to its knees through gross economic mismanagement. This began in earnest in 2000, when take–overs of white–owned farms were passed onto many of Mr Mugabe’s supporters with no experience of modern farming – causing sharp falls in the country’s main export earner. The central bank began printing money to pay off debts and offset higher prices caused by failing farms, leading to severe hyperinflation. At one point, the 100 trillion Zimbabwean dollar was worth US$ 0.40, and ultimately was abandoned, to be replaced by a multi–currency system dominated by the US$. A brief power–sharing arrangement with the country’s opposition tempered some of Mr Mugabe’s destruction, but this ended in 2013 with his party’s resumption of full control. Behind the scenes, the struggle to succeed the 93–year–old president has begun, but without a designated successor the potential vacuum can only add to the country’s woes. (Economist, 27 February 2017)

➢ Intensive animal–raising techniques, where animals are crammed together in small spaces, means that antibiotics are routinely given to healthy animals to prevent the spread of infectious diseases, and to fatten animals. As the demand for meat is set to rise in the large emerging economies, it is estimated that their use will double over the next 20 years, while bacteria evolve resistance to antibiotics. Some experts estimate that drug–resistance bacteria could kill 10 million people by 2050, at a cost of US$ 1 trillion. In economic terms, the use of antibiotics in this context is an example of “the tragedy of the commons”, whereby individuals pursuing their own interests ultimately create a collective disaster. Until the 1970s, new antibiotics were regularly discovered to replace those to which resistance had evolved, but new developments have since dried up. It can be argued that the real solution lies in giving drug companies better incentives to develop new antibiotics, such as “advanced market commitment”, where donors promise to pay for drugs that do not yet exist, as well as tighter regulation of the use of antibiotics and improved animal welfare to discourage the spread of disease. (BBC, 6 March 2017)

➢ China has more than 2000 mobile health care apps, covering medical advice, appointment booking and niche services, and tapping into demand by offering convenience in country where clinics are widely distrusted and hospitals are overcrowded as a result. They appeal to consumers who are underserved by government–run facilities, with inadequate access to doctors, particularly in rural areas. These apps cannot charge for booking hospital appointments, and so rely on advertising and referrals to doctors – which can be charged for – to generate income. However, investors are beginning to lose confidence in these companies profitability, and investment dropped by 10% year–on–year in the last quarter of 2016, after a 41% decline in the previous quarter, despite burgeoning user numbers. In line with China’s ecommerce sector movement toward bricks and mortar retailing, medical apps are finding tie–ups with hospitals essential to boost revenues, and WeDoctor aims to set up 100 hospitals over the next 3 years; and another app is diversifying into private health insurance. Others have expanded into consumer services, such as over–the–counter medicines or cosmetic procedures like Botox. This move toward diversification is underpinned by fears that medical apps will face further regulations, following the investigation of advertisements on Baidu’s search engine for private hospitals offering unnecessary treatments. (Financial Times, 9 March 2017)

➢ Myanmar’s government is considering a change to the law to allow foreign investors to take stakes of up to 35% in the country’s banks. Myanmar’s banking system is one of the most under–developed in the world, and is unable to support the country’s aspiration for fast and inclusive economic development. However, it is one of the fastest–growing banking sectors in Southeast Asia, albeit starting from a very low base. Moreover, the World Bank recently announced plans to work with Myanmar’s government to carry out the first audit of the country’s banking system in decades, in an attempt to modernise it, and to support economic growth. (Irrawaddy, 13 March 2017)

### Energy

➢ Solar power provides a mere 1.3% of the US’s electricity, but the labour–intensive nature of the technology’s design and manufacturing, plus marketing and installation, means that the industry employs more than 260 000 people. And with the solar industry generating 1–in–50 of all new jobs, it is growing quickly. This is slightly more people than in the natural gas industry, over twice as many as the coal industry, over three times as many as wind energy, and almost five times the number in nuclear energy; and only the oil/petroleum sectors employ more people. Although solar power is growing from a low base, and is in a labour–intensive period of its growth, it is worth considering the implications of these statistics. First, although the jobs growth is welcome, it may keep the solar power prices high, as the industry is labour–intensive. Ultimately, this could deter the spread of clean energy, and may need to be addressed by economies–of–scale and automation. Second, the solar industry still lacks the political leverage of the coal or petroleum industries. It is also relatively concentrated in certain states (eg, California), although it is beginning to fan out across the US. President Trump has previously criticized solar power and the White House energy paper makes no mention of it; but as the industry grows and adds jobs, it will become harder to ignore. (Vox, 7 February 2017)

➢ According to a report from the Energy and Resources Institute (TERI) in New Delhi, India will not need to build another coal plan after 2025 if renewables continue to fall in cost at their current rate. This also suggests that its carbon levels could be cut beyond the levels agreed at the recent climate talks in Paris, reducing CO_2_ emissions by about 600 million tonnes, or 10%, after 2030. This is significant, because India is the world’s fastest–growing major polluter and the third–largest emitter of CO_2_ behind China and the USA, and its ability to curb carbon emissions is vital in capping the rise in global temperatures. Much of its growth in CO_2_ emissions is driven by increased electricity consumption – 60% of which is provided by coal–fired plants. India plans to build an extra 65 gigawatts of coal–fired capacity in the next few years, although it already has 308 gigawatts of capacity, with 156 gigawatts being the highest amount ever used. According to TERI’s research, the coal–fired plants under construction will be built, but no more will be needed after 2025 provided that these conditions are met. First, renewables and battery storage prices must keep falling – if they reduce to 50% of their current prices by 2025, they will be cheaper than coal – entirely feasible at their current rates. Second, the government needs to adopt policies to make it possible for electricity companies to switch quickly between renewables and storage energy, eg, if solar power generation is disrupted by weather conditions, a company should be able to instantly buy replacement power from a stored source, such as batteries. (Financial Times, 22 February 2017)

➢ Nigeria’s parliament voted to pass its 2017 budget more swiftly than previous years, to avoid delays in signing the spending plans into law. President Muhammadu Buhari presented the US$ 23.2 billion budget aims to pull Nigeria out of its first recession for 25 years. The recession was caused by low oil prices and attacks on energy facilities in the Niger Delta oil hub. Financial pressures require Nigeria’s government to control the wide–scale corruption and loose tax policy that allows many to siphon off the country’s oil production, hide their wealth and avoid paying taxes. According to government figures, only 214 people pay US$62 232 in taxes, despite a high number of extremely wealthy people who benefit from the country’s oil reserves, and is one of the lowest rates of taxation in the world. (Reuters UK, 30 March 2017)

➢ Peat, despite aromatically flavouring whiskey, and being agreeable and cheap to burn, is one of the dirtiest fuels in use, emitting 23% more CO_2_ than coal. It has been used for fuel in Ireland for at least 1000 years, and today produces 6% of the country’s energy – indeed, Ireland is unusual among developed countries by burning it on an industrial scale. However, Ireland is beginning to turn to other energy sources, including another it also possesses in abundance – wind. Galway Wind Park, scheduled to open later in 2017, will generate 169MW of power at its peak capacity – 3% of Ireland’s average needs. This is the latest development in Ireland’s growing use of wind power, tripling over the past 10 years to reach 3GW of capacity, and overall renewable energy accounts for 25% of Ireland’s electricity consumption, and further wind capacity is being planned. Wind power is difficult to manage and unpredictable, and other countries export excess power that takes their grids beyond the point of stability. Ireland already has two connections to the UK, and the Irish power company, Eirgrid, is planning to expand energy exports into continental Europe. Ireland could potentially meet its entire domestic energy demand from wind power by 2030, with surplus for export – and Bord na Móna, the body responsible for Ireland’s peatlands, plans to stop cutting peat for energy in the same year. (The Economist, 22 April 2017)

➢ In April, Germany set a new record for renewable energy, as at one point energy produced from renewable sources nearly obliterated coal and nuclear power, producing nearly 85% of the country’s total energy. Germany, under its Energiewende initiative, is moving away from fossil fuels and nuclear energy, and plans to transition to low–carbon energy generation by 2050. The success of this policy is shown by April’s record – on 30 April, electricity prices fell into negative figures as renewable sources fed so much power into Germany’s grid that supply exceeded demand. Coal use also fell to a record low at the same time, producing under 8 GW of energy – well under their maximum output of 50G, and most plants were only operational between 3–4 pm. Germany’s nuclear power plants – which will be phased out by 2022 – were also operating at reduced capacity. Germany’s Energiewende plan requires that at least 80% of all power to be generated from renewable sources by 2050, with intermediate targets of 35–40% by 2025; and 55–60% by 2035. (The Independent, 5 May 2017)

### Environment

➢ According to the environmental organization International Rivers, Laos’s plan to push ahead with its Pak Beng dam on the Mekong River, along with related projects, could cause 6700 people to be re–located, and 25 villages in Laos and 2 villages in Thailand being directly affected. This is contrary to government claims that only 1000 people will be affected, all of whom will be compensated. This project is part of a proposed cascade of 11 dams on the main river, but environmental experts claim that building so many dams on the Mekong River could turn it into a series of interconnected lakes, affecting fisheries, sediment and hydrological flows. Most of the 912 megawatts of energy generated by the dam would be exported to Thailand. Dr Daovong Phonekeo, director–general of Lao Ministry of Energy and Mines, is confident that the project’s side effects are solvable, and points out that all development projects have side–effects. Dr Phonekeo believes that the project will turn Laos into a major power exporter and become the “battery for Southeast Asia”. “The Lao government has already decided to go ahead with the project because it is a good project. It will turn water into a useful resource instead of letting water flow down the river uselessly. We want to make this resource more valuable,” he said. (Radio Free Asia, 27 February 2017)

➢ President Donald Trump has signed an executive order which nullifies former President Barack Obama’s Clean Power Plan, which would have closed hundreds of coal–fired stations, frozen the construction of new plants and replaced them with new wind and solar farms. The Clean Power Plan aimed to curb greenhouse–gas pollution from coal–powered power plants, and President Trump’s election campaign made it clear that jobs in the energy industry had higher priority over the global campaign against climate change. Barack Obama had pledged to cut US greenhouse gas emissions by 26% from their 2005 levels by 2025, and the Clean Power Plan was an essential part of this strategy. Although Mr Trump has not formally withdrawn from the Paris Agreement, which aims to keep global warming within 3.6°C and avoid catastrophic climate change, the US, the world’s second–largest polluter, has signaled its non–compliance. (New York Times, 28 March 2017)

➢ Anne Hidalgo and Sadiq Khan, the mayors of Paris and London respectively, announced schemes to score new vehicles on their emissions and impact on air quality. This will enable car buyers to identify the most environmentally–friendly models and chose cars that will reduce pollution. Existing scoring schemes only cover some pollutants and require vehicles to meet standards in laboratory conditions only, when actual road emissions can be up to 15 times higher. The new “cleaner vehicle checker” will allocate each model with a score, based on all pollutants released during on–road conditions, and will more clearly and accurately detail actual emissions. The scheme aims to restore public confidence, after many motorists bought cleaner cars, only to find out that their pollution was much higher because manufacturers used “cheat devices”, or there were flaws in the testing process. Worldwide, other cities, including Seoul, Madrid, Mexico City, Milan, Moscow, Oslo and Tokyo have committed to work with partner cities to develop a global scoring system which is relevant and accessible to all citizens. “For too long, some vehicle manufacturers have been able to hide behind inconsistent regulation and consumer uncertainty about the damage their cars are causing. This announcement is a wake–up call to car companies that they need to act now,” said Mayor Hidalgo. (Cities Today, 13 April 2017)

➢ Global emissions of greenhouse gases have been stable for the past three years, helped by China’s 4–year economic slowdown. However, China’s economic growth picked up, growing at an annualised rate of 6.9% in the first quarter of 2017 – its fastest rate in 18 months. This welcome economic revival has led to increased smog levels in northern China and the southern manufacturing areas, after 3 years of improved air quality and reduced coal consumption. China’s greenhouse gas emissions fell by 1% in in 2016, which combined with lower US emissions, had helped stabilize global emissions. However, official data shows that key industrial areas have suffered marked deterioration in air quality, with PM2.5 levels 32% higher compared to 2015 (PM2.5 particulates are particles with a diameter less than 2.5 µm). This data raises questions on whether China’s stabilising emissions are a result of policies to clean up its air quality and comply with international agreements on climate change; or simply the result of weak economic growth. Moreover, the falling demand for coal has triggered the diversification of coal into gas, which helps China meet its goals for gas usage, but without reduction in coal dependency. If the projected coal–to–gas plants are built, China’s carbon emissions would increase by 1.5% each year. (Financial Times, 26 April 2017)

➢ Every year, illegal fishing removes an estimated 26 million tonnes of seafood from the world’s oceans, worth an estimated US$ 26 billion. This is compounded by its linkages with human trafficking and labour exploitation, which support illegal fishing activities. Illegal fishing gravely threatens the environment, livelihood and health of the Pacific Island nations, whose ocean resources are already threatened by climate change. The problem was considered by the World Economic Forum’s ASEAN conference, and several obstacles to tackling illegal fishing were highlighted. First, the lack of prosecutions and convictions over human trafficking are no threat to the industry’s value, and strong government action is the only way to combat this. Increased co–operation between countries to check boats, limit fishing permits, and revoke licenses if any breaches of the law occur is also vital. The recent Tuna Traceability Initiative is an example of how this can work in practice – organizations must co–operate to converse and manage fish stocks, including companies fully disclosing their environmental management processes and ensure that no slavery is used in the supply chain. (Devex, 5 May 2017)

### Food, Water and Sanitation

➢ In an effort to tackle obesity, France has banned restaurants from offering unlimited sugary drinks, either at a fixed price or for free. France’s rate of overweight or obese adults (15.3%) is below the EU average (15.9%), but is rising, and past the age of 30, nearly 50% of French men and 41% of French women are overweight or obese. The move will affect all public eateries, from fast–food restaurants to school canteens, and targets soft drinks, including sports drinks containing added sugar or sweeteners. The WHO recommends that sugary drinks are taxed, partly due to their links with obesity and diabetes. Elsewhere, a 10% tax on soft drinks in Mexico reduced consumption by 6% in its first year, and the UK will introduce a soft drinks tax in 2018. However, a court blocked an attempt in New York to ban “super–sized” sugary drinks in 2013. (BBC, 27 January 2017)

➢ According to the South Sudan government and three UN agencies, famine has been declared in parts of South Sudan – the result of the country’s protracted civil war and devastating economic crisis. According to UN officials, President Salva Kiir’s government is blocking food aid to some areas. More than 100 000 people in two counties of Unity State are affected, with fears that famine will spread to an additional 1 million people, as 1–in–3 households in South Sudan face food insecurity, and nearly 75% have inadequate food supplies. Widespread hunger has been worsened by South Sudan’s economic crisis, where crippling inflation makes food unaffordable for many families. Providing humanitarian aid is hampered by fighting between the government and armed groups, coupled with the affected population’s inaccessibility – 70% life in the bush. However, UN official Joyce Luma highlights how much of the famine is man–made. “There is only so much that humanitarian assistance can achieve in the absence of meaningful peace and security,” she says. (Al Jazeera, 21 February 2017)

➢ The spread of quinoa consumption in developed countries illustrates how increasingly people are eating unfamiliar grains, as westerners eat less wheat and more millet, sorghum, teff and quinoa, and middle–class Asians eat more wheat in place of rice; and West Africans eat 25% more rice per head than 2006, while millet consumption has fallen by the same amount. These trends show increased prosperity and expanding choice, as better farming techniques improve yields, and rapid urbanisation means fewer people growing their own grains, but have the money to try new varieties. And globalisation means that food and farming techniques cross borders, enabling more people to try new flavours and foodstuffs. This is part of a broader picture of falling hunger levels – between 1990 and 2015, the proportion of malnourished children fell from 25% to 14%, the proportion of income that poor people spend on food fell from 79% to 54%, and among under–nourished people, the average calorie shortfall fell from 170/d to 88/d by 2016. In light of these benefits of globalisation, Donald Trump’s plans to erect trade barriers and possibly start a trade war give food for thought. (Economist, 9 March 2017)

➢ According to the UN’s World Water Development Report, globally more than 80% of wastewater is discharged untreated into rivers and lakes, with negative consequences for health and the environment. However, according to the report’s editor–in–chief, Richard Connor, wastewater is a resource which could help meet the water, energy and nutrient needs of a growing global population. Wastewater contains nutrients such as phosphorus and nitrates which could be turned into fertiliser, and treated sludge can be turned into biogas that could power wastewater treatment plants. The UN estimates that the world will need 55% more water and 70% more energy by 2050 to meet global population growth, and more people also means more wastewater. Dealing with wastewater is also a huge challenge within informal settlements in rapidly–growing cities in developing countries. He calls for governments to invest in smaller, decentralized treatment systems that are cheaper and easier to maintain, and notes that not all water needs to be treated to drinking quality, but to a level where it can be used by industries, municipalities, agriculture or for cooling in power plants. (Thomson Reuters Foundation, 22 March 2017)

➢ More than 1–in–5 of South Africa’s children suffers from stunted growth, and according to the 2016 Global Nutrition Report, South Africa ranks 70 out of 132 countries on this indicator. It performs slightly worse than several poorer African countries, including Gabon, Ghana and Senegal, and only slightly better than many others, including Somalia and Swaziland. There has been only a slight reduction in stunting among South Africa’s children over the past 20 years, and the country’s system of social grant payments is doing little to tackle the problem. South Africa’s parliament approved a slight increase to these grants, and the Child Support Grant covers 12 million children – almost 66% of all children in South Africa. Despite this coverage, the grants are not tackling malnutrition, partly because any increases in their value have been outstripped by food price inflation, and they cover less than two–thirds of the cost of a providing a nutritionally–adequate diet. Second, the grants are often partially spent on non–food needs for the household as a whole. Third, the grants do not tackle the other causes of malnutrition in South Africa, including unsanitary water supplies leading to diarrhea or worms; and the low number of women breast–feeding their babies, which can damage a child’s nutritional status from birth onwards – South Africa has one of the lowest compliance with WHO breast–feeding recommendations in the world. (The Conversation, 2 April 2017)

### Peace and Human Rights

➢ Women and girls in Afghanistan can face imprisonment of up to 5 years in the country’s so–called “moral prisons”, for crimes such as running away from home (even if fleeing violence), or for sex before marriage (*zina*). According to the campaigning group Human Rights Watch, these incarcerations are increasing, from 400 in 2011 to 600 in 2013. The Afghan government has consistently rejected abolishing the prosecution of women for moral crimes. Women imprisoned for moral crimes have described imprisonment without trial, or on false charges, or for *zina* despite being raped. Afghanistan also permits the use of “virginity tests” – with or without the consent of the woman or girl concerned – for those who have fled their homes or entered public spaces without male supervision. Conditions inside the prisons are harsh, with inadequate ventilation, space and sanitation. Many inmates give birth inside prison, or have their children with them during their imprisonment. After their sentences, women are transferred to secret shelters to protect them from honor killings. Afghanistan’s justice system favors men, with a reported 5132 new cases of violence against women (including 241 murders) in the first half of 2016 – in almost all cases, the perpetrators were unpunished. In addition, informal courts, chaired by powerful male fundamentalists, have carried out public lashings and executions against women, and in the past the Afghan government has attempted to make stoning legal for certain violations of Sharia law, such as adultery. It has also failed to fully implement the country’s 2009 Elimination of Violence Against Women law – a presidential decree which aimed to protect the rights of women and girls. (The Diplomat, 8 March 2017)

➢ According to sources in North Korea, women serving in the country’s military and construction brigades are routinely abused by their supervisors, and some are pressurised into providing sexual favors and forced into sex work. North Korea’s semi–military construction brigades, known as “storm troopers” will assign women to the most difficult workplaces if they reject their commanders’ sexual demands. Moreover, women cleaning up after the floods in Yonsa country could not bathe at night despite being covered in mud, because border guards prevented them from approaching the Tumen River. “Although equal rights for both sexes have been guaranteed in North Korea for more than 70 years, it is hard to find another country in which women’s rights are trampled this badly,” an unnamed source said, calling for compulsory service in the army and construction brigades to be immediately abolished to safeguard women’s rights. (Radio Free Asia, 10 March 2017)

➢ Police have used tear gas and bullets to disperse protesters who had gathered in Kinshasa, the capital of the Democratic Republic of Congo, and a number of injuries have been reported. Protests began when negotiations over the departure of President Joseph Kabila after 17 years in power collapsed – an outline peace deal was agreed in 2016 but has proved difficult to finalise. Church leaders had mediated talks between the government and opposition, but have withdrawn after both sides failed to agree on issues such as the choice of a transitional prime minister. Mr Kabile’s term ended in December 2016, and the opposition has accused the government of sabotaging efforts to offer him a peaceful exit. The main opposition party, the Union for Democracy and Social Progress, has called on citizen to take part in a peaceful march on 10 April, to “resist the dictatorship taking root.” (BBC, 29 March 2017)

➢ Despite tensions over other policy areas, global health is emerging as a potential area of partnership between the USA and China. Both countries collaborated in the SARS outbreak in 2002, and Ebola in 2014, when staff at the US Center for Disease Control and Prevention (CDC) worked with staff at Chinese laboratories. This partnership is ongoing, as China and the US’s CDCs work to build an African CDC to combat infectious diseases, and is expanding into the private arena. This is illustrated by the Bill and Melinda Gates Foundation part–funding the Global Health Drug Discovery Institute in Beijing – the first foreign–funded NGO to operate in China, which will focus on early drug discovery to combat infectious diseases such as TB, malaria and HIV. This evolving role is part of China’s long–term strategy of building up its pharmaceutical industry, and becoming a key player in global health, as it moves from being an aid recipient to an aid provider. During the Ebola outbreak, Chinese scientists reverse–engineered the US/Canadian drug, ZMapp, after the manufacturers exhausted their limited supplies – a key indicator of China’s role in international health crises. Moreover, the potential reduction in US aid contributions under the Trump presidency (the US is currently the largest global health funder, with an estimated US$10.2 billion of aid in 2016), creates a potential void that China could be positioned to fill. (Forbes, 26 April 2017)

➢ Several years after civil war in the Central African Republic (CAR) killed thousands of people and left hundreds of thousands of people displaced, aid workers have warned that the country may be returning to conflict. In recent months, armed groups have killed at least 45 people and burned villages, and more than 100 000 people have fled their homes. The conflict is mainly between mainly Christian rebels (anti–balaka) and the mostly Muslim former Séléka rebels. Anti–balaka rebels had used the village of Bambara as a base in northern CAR; the village was then attached by former Séléka rebels, killing 25 people and burning 600 homes. The surviving villagers have no food or seeds, and lack clean water and education, as the school was also destroyed. About 20% of the CAR’s population – about 400 000 people – are displaced, and according to the agency Médecins sans Frontières (MSF), civilians are being attached at levels not seen in years. Since the civil war, more than 50% of the population rely on humanitarian aid, but aid levels are only 10% of what is required. According to the UN, this lack of support further damages any chance of peace. MSF are experiencing severe difficulties in reaching distant rural areas in need. “CAR is one of the poorest countries in the world, and needs to be supported but the people are focusing on the conflict. But it takes time for people to solve it. During this time, we should be able to carry on with normal activities to give this access and it’s not easy,” says Abdel Kader Tlidjane of MSF. (Voice of America, 6 May 2017)

### Science and Technology

➢ Despite many tragedies in 2016, such as the bombing of hospitals in conflict zones, the increasing threat of Zika and antibiotic–resistant microbes, the re–emergence of polio in Nigeria and the revival of yellow fever, there were many inspiring developments within global health. One of these was the continuing progress in combating malaria – Africa, which has the highest mortality rates from malaria, saw a fall in deaths from 800 000 in 2000 to 400 000 in 2016. In addition, European drug regulators approached the first human vaccine against malaria – although its protection weakens over time, it is still a major breakthrough. 2016 also saw the approval of the first vaccine against dengue fever, which causes nearly 50 million infections a year and is the world’s most significant and fastest–growing mosquito–borne viral disease. HIV infections and deaths have stabilized, the Americas are almost free from river blindness and other tropical diseases nearing elimination include lymphatic filariasis and guinea worm. An experimental Ebola vaccine has been produced, and although it is not yet approved an emergency stockpile of 300 000 doses has been created in the event of another outbreak. (Project Syndicate, 17 January 2017)

➢ An outbreak of yellow fever in Angola in 2016 infected more than 7000 people and caused hundreds of deaths before it was brought under control. Yellow fever is vaccine–preventable, but the outbreak highlighted that the WHO’s supply of 6 million doses was inadequate – catastrophe was averted by international co–operation, science and luck, as drought reduced the population of yellow–fever carrying mosquitoes. It shows the fragile state of the world’s systems for responding to dangerous pandemics, because only a few companies manufacture yellow–fever vaccine, thanks to unstable markets and uncertain profits, and if yellow fever had spread into China by returning migrant workers, demand would have far exceeded global manufacturing capacity. The new Coalition for Epidemic Preparedness Innovations (CEPI) exists to facilitate the development of vaccines for threatening diseases, and to build capacity to respond when new diseases emerge, and is an important milestone in epidemic preparedness and prevention. However, effective pandemic preparedness depends on our ability to connect innovations from R&D to the logistical capacities to deliver supplies where they are needed. Local surveillance capacities, laboratory capacity, diagnostic tools and health information systems must also be strengthened, and emergency operations centers established to improve responses, plus training health workers in digital technologies. Strengthening every link in the chain of epidemic preparedness and response is vital to ensure that the next outbreak of disease does not wreck the same devastation as Ebola, Zika or yellow fever. (Financial Times, 27 January 2017)

➢ The ability to use drones to sample animals and people to determine which pathogens are present in an area and where they are hosted would be invaluable in understanding how diseases spread, and how to predict and pre–empt their outbreaks. Drone technology is not yet sufficiently advanced to allow this, but scientists at Microsoft Research have designed a system that captures mosquitoes and analyses their pathogen load from drawing blood from feeding off their hosts, thereby detecting blood–borne pathogens present in the host other than those transmitted by mosquitoes, eg, malaria. Ethan Jackson and Jonathan Carlson of Microsoft Research designed portable mosquito traps that lures in the insects (the traps can be fine–tuned to detect mosquitoes of certain species). The captured insects are extracted, triturated and their collective DNA analyzed and matched against a database of known sequences, potentially also identifying unknown viruses. Ultimately, it is hoped to produce traps that can be carried, deployed and collected by drones in inaccessible areas, which are home to wild animals that act as reservoirs for pathogens like Ebola that can spread to humans. (Economist, 23 February 2017)

➢ Each day, 1300 children die of diarrhea, and rotavirus causes 33% of these deaths, making it the second–largest cause of death among children and babies. Rotavirus is vaccine–preventable, but most of these children live in sub–Saharan Africa, with massive problems in keeping vaccines cold enough during transit and storage. However, a new heat–stable rotavirus vaccine, BRV–PV, has been developed and a Phase II study found that it protects against gastroenteritis in 66% of children who receive it. A trial was conducted in Niger, which has a population of 20 million people, half of whom aged under 15, and the majority living in poverty. It is also land–locked and largely desert, with little access to electricity and water, and with most people living far from health centers. BRV–PV outperformed both existing vaccines in the trial, and is being reviewed by the WHO for pre–qualification, which would make it available in low–income countries. A heat–stable vaccine offers hope for reaching more children, and the cost, at US$ 2.50/dose, is lower than both existing vaccines that require refrigeration – making a huge difference to the lives of children and their families. (Forbes, 23 March 2017)

➢ The Democratic Republic of the Congo (DRC) is moving toward using an unlicensed vaccine against an outbreak of Ebola in a remote area. The WHO has issued a “donor alert”, requesting US$ 10.5 million to support the vaccine trial, including surveillance, treatment, and conventional prevention and control techniques. The DRC government submitted a formal vaccine trial protocol to an ethical review board. The vaccine was produced by Merck and stockpiled in the USA after the 2014 outbreak in West Africa. The WHO and Médecins sans Frontières (MSF) set up vaccine trial in Guinea, with an unusual “ring vaccination” design that selectively vaccinated those who were most likely to have had contact with a known case. The initial results showed 100% protection 10 days after immunisation, but the unusual trial design dissuaded Merck from pursuing it further, and the vaccine can still only be used in trial setting. Epicenter (MSF’s research arm) and DRC’s Ministry of Health have written a protocol for a new ring vaccination study, but without a control group because withholding the vaccine from some participants is no longer seen as ethical. However, this means that the trial cannot evaluate its efficacy. (Science, 24 May 2017)

